# Immune hyporeactivity to bacteria and multiple TLR-ligands, yet no response to checkpoint inhibition in patients just after meeting Sepsis-3 criteria

**DOI:** 10.1371/journal.pone.0273247

**Published:** 2022-08-18

**Authors:** Alexandra Bick, Willem Buys, Andrea Engler, Rabea Madel, Mazen Atia, Francesca Faro, Astrid M. Westendorf, Andreas Limmer, Jan Buer, Frank Herbstreit, Carsten J. Kirschning, Jürgen Peters

**Affiliations:** 1 Klinik für Anästhesiologie und Intensivmedizin, Universität Duisburg Essen & Universitätsklinikum Essen, Essen, Germany; 2 Universität Duisburg-Essen, Essen, Germany; 3 Institut für Medizinische Mikrobiologie, Universität Duisburg Essen & Universitätsklinikum Essen, Essen, Germany; Hamad Medical Corporation, QATAR

## Abstract

**Rationale:**

The immune profile of sepsis patients is incompletely understood and hyperinflammation and hypoinflammation may occur concurrently or sequentially. Immune checkpoint inhibition (ICI) may counter hypoinflammation but effects are uncertain. We tested the reactivity of septic whole blood to bacteria, Toll-like receptor (TLR) ligands and to ICI.

**Methods:**

Whole blood assays of 61 patients’ samples within 24h of meeting sepsis-3 criteria and 12 age and sex-matched healthy volunteers. Measurements included pattern/danger-associated molecular pattern (P/DAMP), cytokine concentrations at baseline and in response to TLR 2, 4, and 7/8 ligands, heat-inactivated *Staphylococcus aureus* or *Escherichia coli*, *E*.*coli* lipopolysaccharide (LPS), concentration of soluble and cellular immune checkpoint molecules, and cytokine concentrations in response to ICI directed against programmed-death receptor 1 (PD1), PD1-ligand 1, or cytotoxic T-lymphocyte antigen 4, both in the absence and presence of LPS.

**Main results:**

In sepsis, concentrations of P/DAMPs and inflammatory cytokines were increased and the latter increased further upon incubation *ex vivo*. However, cytokine responses to TLR 2, 4, and 7/8 ligands, heat-inactivated *S*. *aureus* or *E*. *coli*, and *E*. *coli* LPS were all depressed. Depression of the response to LPS was associated with increased in-hospital mortality. Despite increased PD-1 expression on monocytes and T-cells, and monocyte CTLA-4 expression, however, addition of corresponding checkpoint inhibitors to assays failed to increase inflammatory cytokine concentrations in the absence and presence of LPS.

**Conclusion:**

Patients first meeting Sepsis-3 criteria reveal 1) depressed responses to multiple TLR-ligands, bacteria, and bacterial LPS, despite concomitant inflammation, but 2) no response to immune checkpoint inhibition.

## Introduction

Systemic inflammation and host damage in sepsis rely on mediators evoked by the interaction of pathogens, pathogen associated molecular patterns (PAMPs), and danger associated molecular patterns (D)-AMPs [1] with cognate pattern recognition receptors such as toll-like receptors (TLR).

While insights into mechanisms of sepsis using animal experiments and cells are copious, comprehensive studies of mechanisms in patients are rather scarce. Translation is hampered by different mechanisms or receptor repertoires in the response to bacteria [2, 3], changing sepsis definitions over time [4], uncertainty about study inclusion criteria and timing of samples [5], clinical difficulties of specimen sampling on a fixed time scale, or confounders such as high dose corticoid therapy [6] and the potential impact of genetic effects such as single nucleotide polymorphisms [7–9]. To ease some of these difficulties and investigate more homogenous cohorts, the Sepsis-3 definition has been proposed [10]. It is largely unknown, however, what kind of immune profile patients show when just meeting Sepsis-3 criteria and whether this relates to prognosis. According to common views early sepsis is dominated by hyperinflammation while a compensatory “anti-inflammatory response syndrome” (CARS) [11], “immunoparalysis” [12], or “immune reprogramming” [13] may surface later. Other investigators, however, have argued that immunosuppression occurs earlier [5, 14] and suggested immunostimulatory treatments, shifting interest from dampening hyperinflammation to counteracting “immunoparalysis” via immune checkpoint inhibition (ICI) [4, 15] analogous to their success as cancer treatment [16].

Accordingly, we assessed in whole blood assays from patients first meeting Sepsis-3 criteria, in addition to measurements of P/DAMP concentration and blood-intrinsic inflammatory activity, the response to added TLR 2, 4, and 7/8 ligands, heat-inactivated *S*. *aureus* and *E*. *coli*, *E*. *coli* lipopolysaccharide, and several immune checkpoint inhibitors in the absence and presence of LPS.

Specifically, we tested the hypotheses that 1) inflammatory cytokine responses to multiple TLR-ligands, Gram-positive and Gram-negative bacteria, and LPS are already depressed during the first 24 hours of sepsis and 2) ICI sparks cytokine secretion.

## Material and methods

### Septic patient and control cohorts, clinical data collection, and blood sampling

Following ethics committee approval (Medical Faculty, #17-7330-B0) of this prospective observational study (2018–2019), Intensive Care Unit (ICU) patients of the Universitätsklinikum Essen were screened for early sepsis using the Sepsis-3 criteria [10], i.e., showing within the last 24 hours an increase in the sepsis-related organ failure assessment (SOFA) score by ≥2 points and suspected infection. Patients meeting Sepsis-3 criteria already for more than 24 hours, those under 18 years of age, on immunosuppressive medications (taking over 30 mg/d cortisol equivalent), or with HIV infection were excluded. Blood from 61 patients that had been drawn via central catheters into tubes containing 16 IU/ml unfractionated heparin (02.1064, Sarstedt, Nümbrecht, Germany) was available and transferred to our laboratories at room temperature within 20–30 minutes. Medical history, clinical status, medication, hemodynamic, hematological, and laboratory data (Table 1), results from cultures of blood and other sources, and PCR (SeptiFast, Roche, Basel, Switzerland) for DNA typical for 20 frequent pathogens (S1 Table) were documented. SAPS-II and SOFA-Scores were calculated, and patients assessed for the presence of septic shock, positive qSOFA, and SIRS criteria (Table 1). Twelve healthy adults served as age and sex matched controls following ethics committee approval (#17-7869-B0) and informed consent.

**Table 1:** Characteristics of sepsis patients and age-matched healthy volunteers.

	Sepsis patients	Healthy volunteers
Age [years]	61.5 ± 14.9	56.5 ± 8.5
Sex Men [n; %]	36; 59	7, 54
Women [n; %]	25; 41	6, 46
Heart rate [min^-1^]	104 ± 25	68 ± 6
Systolic arterial pressure [mmHg]	108 ± 20	134 ± 14
Diastolic arterial pressure [mmHg]	53 ± 13	83 ± 5
Mean arterial pressure [mmHg]	72 ± 14	100 ± 6
Leukocyte concentration [/nl]	20.7 ± 17.4	6.6 ± 1.1
Monocyte concentration [/μl]	569 ± 99	532 ± 51
IL-6 [pg/ml]	108.4 [29.1 | 266.7]	1.0 [0.76 | 1.06]
TNF [pg/ml]	5.8 [4.0 | 10.3]	2.1 [0.33 | 3.48]
IL-10 [pg/ml]	4.3 [1.2 | 14.0]	0.9 [0.36 | 1.06]
IL-8 [pg/ml]	43.8 [19.6 | 142.6]	7.9 [4.12 | 11.83]
IL-1β [pg/ml]	0.7 [0.6 | 1.8]	1.4 [0.72 | 1.44]
IL-1α [pg/ml]	0.6 [0.23 | 2.31]	1.2 [1.06 | 1.27]
Case fatality rate [%]	49	
ICU stay, survivors [days]	31 ± 36	
ICU stay, deceased [days]	16 ± 23	
SOFA-Score [median]	12 [11 | 14]	
SAPS-II Score [median]	72 [59 | 79]	
≥2 qSOFA criteria [%]	65.6	
≥2 SIRS criteria [%]	82	
Shock^a^ [%]	56	
Patients with i.v. catecholamine therapy [%]	91	
Patients with i.v. catecholamine therapy ≥ 0.1 μg/kg body weight / min [%]	56	
Lactate serum concentration [mmol/l]	3.6 ± 4.1	
Patients on mechanical ventilation (%)	80	
P_a_O_2_/ F_i_O_2_ ratio (mmHg)	184 ± 101	
Platelet concentration [/nl]	198 ± 157	
Bilirubin serum concentration [mg/dl]	2.4 ± 6.2	
Creatinine serum concentration [mg/dl]	1.9 ± 1.6	
Patients under renal replacement therapy [n; %]	15; 25	
C-reactive protein serum concentration [mg/l]	20.3 ± 12.1	
Procalcitonin serum concentration [μg/l]	27.7 ± 89.4	

Data from 61 septic patients within 24 h of meeting Sepsis-3 criteria [10] and 12 age-matched healthy volunteers presented as numbers, percentages, or means ± SD. SOFA, SAPS-II score, and cytokine concentrations presented as median [first quartile | third quartile]. ^a^Definition of septic shock as in [10]; i.e., sepsis with a serum lactate concentration >2 mmol/l and any vasopressor therapy to sustain a mean arterial pressure ≥65 mmHg.

Patient care and laboratory personal were unaware of all (anonymized) data to avoid bias. Since neither patients nor volunteers underwent health related interventions registration as a clinical study was not applicable, as confirmed before enrollment by the German clinical study register.

### Whole blood incubation

Twohundred μl of heparinized whole blood were pipetted to wells of 96-well polystyrene plates (Nunc 262162, Thermo Fisher Scientific, Waltham, MA). Subsequently, inhibitors or stimulants were added, and suspensions incubated for 6 or 22 hours under standard conditions (37°C, humidified atmosphere, 5% CO_2_) upon which supernatant mediator concentrations were measured. Individual assays were run in triplicate and pooled for luminex ELISA (see below).

### Mediator and soluble checkpoint molecule concentrations in blood plasma and blood cell incubation supernatants

Concentrations of proteinaceous inflammatory mediators in blood plasma (before or after whole blood incubation) were measured by multiplex ELISA (Luminex LXSAH-06, R&D Systems, Minneapolis, MN) against a standard curve according to the manufacturer’s instructions. Standards were included in the sets. We chose four cytokines commonly associated with human sepsis (TNF, IL-6, IL-1β and α), IL-8 as major granulocyte attractant, and IL-10 as an important anti-inflammatory cytokine.

Plasma concentrations of the soluble immune checkpoint molecules PD-1, PD-L1, CTLA-4, T-cell immunoglobulin and mucin-domain containing (Tim-) 3, lymphocyte-activation gene (Lag-) 3, cluster of differentiation (CD)137 (4-1BB), CD25, and Galectin-9 were assessed by a similar method (HU Immune Checkpoint Panel 1-S/P, BioLegend, San Diego, CA). Measurements had an intertester and repeat reliability of ≥95%. Lower detection limits ranged between 1 and 5 pg/ml, and were 29 pg/ml for Lag-3 and 41 pg/ml for Galectin-9, respectively.

### Expression of cellular checkpoint molecules

Expression of cellular checkpoint molecules was assessed by flow cytometry. Leukocytes from blood were isolated using an aggregation and gravitational separation method (HetaSep^TM^, STEMCELL^TM^ technologies, Vancouver, Canada) according to the manufacturer’s instructions and stored in BamBanker freezing medium (STEMCELL^TM^ technologies). About 5 x10^6^ leukocytes were later stained on ice for 30 min (staining buffer, BioLegend, San Diego, CA). After washing, cells were taken up in 50 μl staining buffer containing a dead cell detection reagent (Zombie aqua^TM^, BioLegend). Expressions were measured by flow cytometry (Cytoflex s, Beckman Coulter, Brea, CA) and analyzed using a DeNovo software (FCS Express 7, Pasadena, CA). Details on antibodies used, FACS-settings, and gating strategy are given in the S2 File.

### Quantification of circulating host mitochondrial, genomic, and bacterial DNA in blood plasma

Total DNA was isolated from 200 μl of plasma (QIAamp DNA Blood Mini Kit, Qiagen, Venlo, The Netherlands) to serve as template for all real-time quantitative PCRs performed by applying a StepOne Plus PCR System (Applied Biosystems, Thermo Fisher Scientific, Waltham, MA). A melting curve was performed after each run.

For mitochondrial DNA the Mesa Green qPCR MasterMix for Sybr Assay (Eurogentec, Seraing, Belgium) was used. Cycling conditions were: 5 min at 95°C followed by 40 cycles of 3 s at 95°C and 40 s at 60°C. The primers for ATPase 6 (5´-TCCCCATACTAGTTATTATCGAAACCA-3’ and 5’-GCCTGCAGTAATGTTAGCGGTTA-3’) and D-Loop (5’-TGCACGCGATAGCATTGC-3’ and 5’-AGGCAGGAATCAAAGACAGATACTG-3’) were designed with Primer Express 3.0 Software (Applied Biosystems, Thermo Fisher Scientific). For quantification, serially diluted DNA samples generated from purified specific PCR products (Invisorb Fragment CleanUp Kit, Stratec Molecular, Birkenfeld, Germany) were used as standard in each run.

Human nuclear DNA was quantified by application of the forensic grade human genomic DNA quantification assay (gDNA detection kit, Primerdesign Ltd., Chandler’s Ford, UK) and the Taqman Fast Advanced Master Mix (Applied Biosystems, Thermo Fisher Scientific). Briefly, amplification of a single copy region of non-transcribed DNA was performed with provided primers and a hybridization probe. A standard curve was generated upon serial dilution of a provided positive control template in parallel to each run. Cycling conditions were 2 min at 50°C, 20 s at 95°C, followed by 50 cycles of 7 s at 95°C and 40 s at 60°C.

Bacterial DNA was quantified by application of Eubacteria genesig® Standard Kit and the oasig lyophilised 2x qPCR Master Mix (Primerdesign Ltd.) according to the manufacturer’s instructions to amplify a specific 16S ribosomal RNA gene segment. A standard curve was prepared by serially diluting the Eubacteria positive control template in each run. Cycling conditions were 2 min at 95°C, 50 cycles of 10 s at 95°C and 60 s at 60°C.

### Analysis of sepsis patients’ and control blood plasma samples for their propensity to activate ectopically overexpressed TLR2 read out as NF-κB dependent luciferase reporter gene activation

Luciferase assaying for analysis of TLR2 ligand activity has been described earlier [3]. In short, TLR2 expression and luciferase reporter plasmids were transiently transfected into largely TLR deficient human embryonic kidney fibroblastoid HEK293 cells to assess TLR2 specific P/DAMP driven NF-κB activation [17]. Twenty μl of blood plasma in 80 ml RMPI 1640 was added to individual wells of 96 well cell culture plates, which were incubated at standard incubation conditions for 16 hours upon which cells were lysed. Reporting and control luciferase activities in cellular lysates were measured with a 96-well plate luminescence reader (Orion II, Titertek-Berthold, Bad Wildbad, Germany).

### Experimental interventions in assays

#### Pattern recognition receptor challenges of whole blood samples from sepsis patients and controls

LPS (*E*.*coli* O111:B4 [18], common long chain LPS, TLR2 & 4 ligand; 100 ng/ml, Sigma-Aldrich, St. Louis, MO), LPS (S. minnesota Re595 [19], a LPS devoid of a long O-chain, TLR4 specific; 100 ng/ml, Sigma-Aldrich, St. Louis, MO), P_3_C (bacterial lipoprotein mimicking synthetic lipohexapeptide [20], TLR2 stimulant; 20 μg/ml. EMC microcollections, Tübingen, Germany), R848 (synthetic nucleic acid derivative [18], TLR 7/8 stimulant; 5 μg/ml, Invivogen, Toulouse, France), sterile PBS suspensions of *S*. *aureus* ([21], 3 x 10^5^ cfu/ml, 20231, DSMZ, Braunschweig, Germany) and *E*. *coli* (clinical isolate 30/185 [17], 1 x 10^4^ cfu/ml) served as stimuli.

#### Immune checkpoint inhibition (ICI) in sepsis patients’ whole blood samples in the absence or presence of *E*. *coli* lipopolysaccharide

ICI can unleash activity of cells inhibited by checkpoint molecules. Antibodies against PD-1 (Nivolumab, Bristol-Myers Squibb, New York City, NY, or Pembrolizumab, MSD, Kenilworth, NJ), PD-L1 (Atezolizumab, Genentech, San Francisco, CA), or CTLA-4 (Ipilimumab, Bristol-Myers Squibb) were added at the start of whole blood sample incubation to yield a final concentration of 30 μg/ml (corresponding to those in anti-cancer therapy [16]) and assays incubated for 22 hours. Supernatant mediator concentrations were measured subsequently in the absence and in the presence of *E*. *coli* LPS (see above) added 6 hours after incubation start in concentrations of 1 ng/ml or 10 ng/ml.

### Statistical methods

Figures were designed and statistical analysis was performed using Graph Pad Prism (GraphPad Software, V8.4.3, San Diego, CA). S1 Fig was designed using Biorender.com.

Continuous clinical variables are presented as means ± standard deviation (SD), discrete clinical variables as percentage of the respective cohort or median with quartiles, clinical data were descriptive. Data were analyzed for non-normality using the Anderson-Darling test. Non-normally distributed values are presented as dot plots with medians and quartiles. All hypotheses were set a priori, and statistical tests were two-tailed. We used the Mann-Whitney U test for unpaired and the Wilcoxon matched pairs signed rank test, as well as the Friedman test with Dunn’s multiple comparison corrected post-test for paired data, as appropriate. P-values were corrected for multiple testing using the Holm-Bonferroni procedure with increase of the p-values (instead of lowering the local α-level), and p-values <0.05 after correction were considered statistically significant. The available sample volume limited the number of intra-assay interventions per patient so that not all types of assay interventions/measurements could be performed on samples from all patients. Twelve healthy volunteers where enrolled over the whole study period. As available and deemed appropriate, 4 to 11 control samples per experiment were run in parallel with sepsis patients’ samples.

To screen for variables potentially predicting in-hospital-mortality we initially compared all *ex vivo* data between hospital survivors and hospital non-survivors and tested for significant differences via the Mann-Whitney test. Of these, IL8 at baseline and IL6 after 6 hours of LPS stimulation (IL8[baseline] and IL6[LPS]) significantly differed between hospital-survivors and hospital non-survivors:

**Table:** 

Variable	Hospital-survivors (Median)	Hospital non-survivors (Median)	p-value
IL8[baseline] (pg/ml)	30.24	111.7	0.0035
IL6[LPS] (pg/ml)	5750	3579	0.0229

We then performed a multiple logistic regression (with Graph Pad Prism standard settings) with in-hospital survival as dependent variable and the significant variables of the first step as independent variables. As IL8 at baseline and IL6 after 6 hours of LPS stimulation significantly correlated (p = 0.003, Spearman’s rank correlation), the assumption of independence was violated. We hence eliminated one variable previously assumed to be independent to choose the model with the lower Akaike information criterion corrected for small sample sizes (AICc). This was IL6 [LPS]: AICc = 57.75 for an IL6[LPS] only-model compared to AICc = 77.25 for an IL8[baseline] only-model). We then performed the appropriate Log-likelihood ratio-test (G-squared) and prepared a receiver operating characteristics (ROC) curve. Tjur’s (Pseudo-) R^2^ and the area under the ROC-curve (with 95% confidence intervals) were calculated.

## Results

Please refer to S1 Fig for a concise summary of findings.

### Characteristics of sepsis patients and controls

As expected, inflammatory, hemodynamic, and metabolic markers were abnormal in patients (Table 1) just meeting Sepsis-3 criteria. About a third of sepsis patients’ blood cultures and a sixth of microbiological diagnostic germ DNA PCRs were positive, and pneumonia was the most common focus (S1 Table). Patients were rather sick with a fatality rate of approximately 50% (Table 1). While patients’ and volunteers’ leukocyte concentrations differed, their monocyte concentrations as the major source of inflammatory cytokines did not (Table 1).

### Depressed inflammatory cytokine response to TLR2, 4, and 7/8 ligands

**Fig 1:**
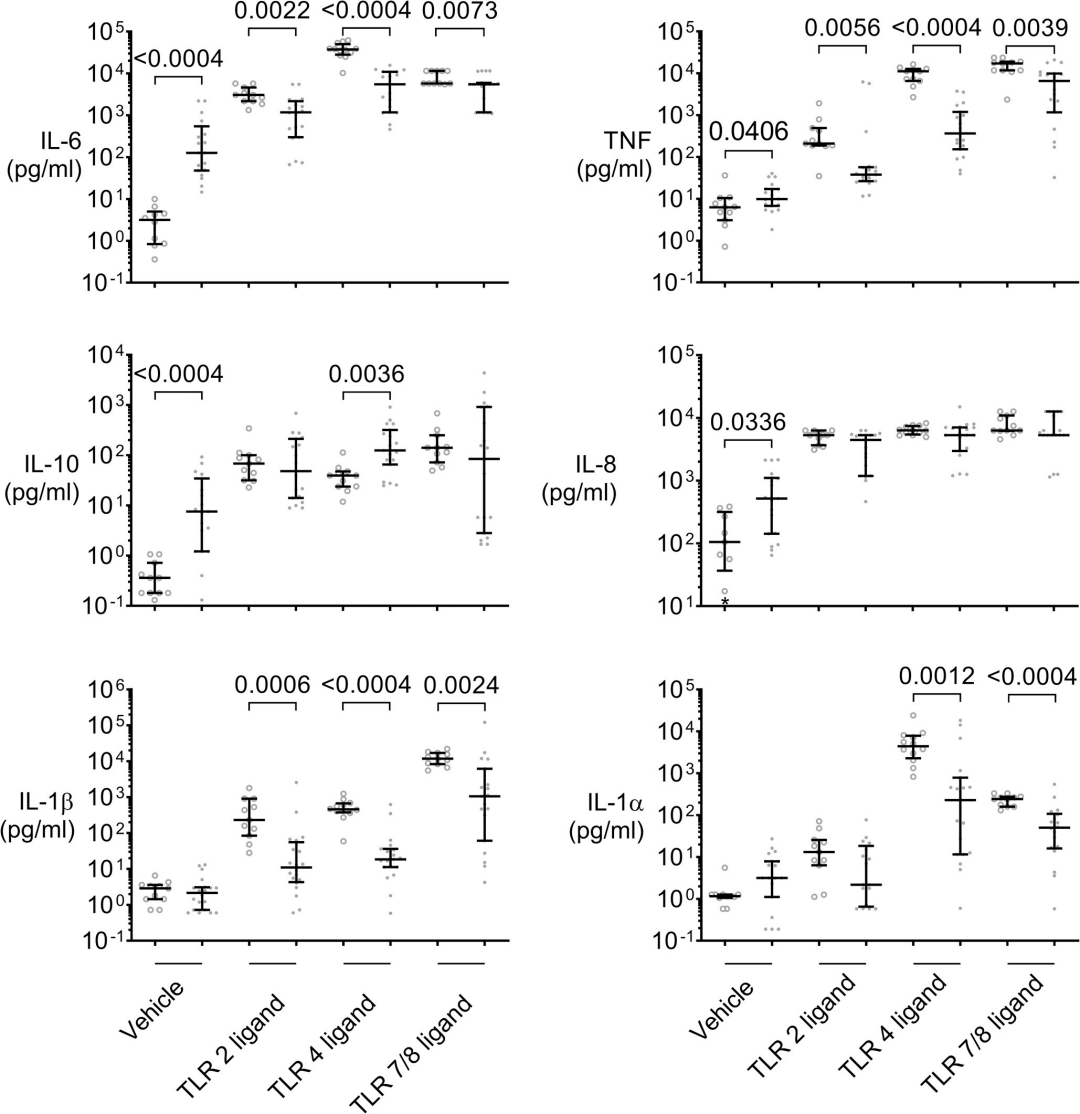
Diminished inflammatory cytokine responses to specific TLR2, 4, and 7/8 ligands in early sepsis patients’ whole blood. Supernatant cytokine concentrations in whole blood assays of 19 sepsis patients (dots) sampled within 24 h of meeting Sepsis-3 criteria and 11 healthy volunteer controls (open circles) incubated for 6 h with either vehicle, TLR2 ligand P_3_C, TLR4-specific ligand LPS Re595, or TLR7/8 agonist R848. Dot plots with median and quartiles. Mann-Whitney tests with Holm-Bonferroni correction for multiple testing. Sepsis patients’ samples show markedly decreased proinflammatory cytokine concentrations (IL-6, TNF, IL-1α and IL-1β) in response to multiple TLR-ligands. In contrast, anti-inflammatory IL-10 is increased with TLR4 stimulation.

Supernatant inflammatory cytokine concentrations upon incubation with TLR 2, 4, and 7/8 ligands increased in samples from both sepsis patients and healthy volunteers compared to vehicle (Fig 1). However, when compared to the concentrations seen in stimulated samples from healthy subjects, this increase was markedly less, although concentrations in sepsis patients at baseline were greater (Fig 1, Table 1). Thus, inflammatory cytokine concentrations in response to multiple TLR-agonists were significantly depressed in assays of sepsis patients compared to healthy volunteers. This applied to IL-6 for the TLR2 ligand (median quotient: 2.6-fold, p = 0.0022), TLR4 ligand (6,8-fold, p<0.0004), and the TLR7/8 ligand (1.05-fold, p = 0.0073) as well as to TNF (5.5–30.6-fold) and IL-1β (11-25-fold) for these ligands (Fig 1).

In contrast, the concentration of IL-10 in sepsis patients significantly increased even beyond values seen in samples from healthy subjects following stimulation with the TLR4 ligand (p = 0.0036), but not significantly following stimulation with TLR2 or 7/8 ligands.

### Depressed cytokine responses to Gram-positive and Gram-negative bacteria in assays of sepsis patients

**Fig 2:**
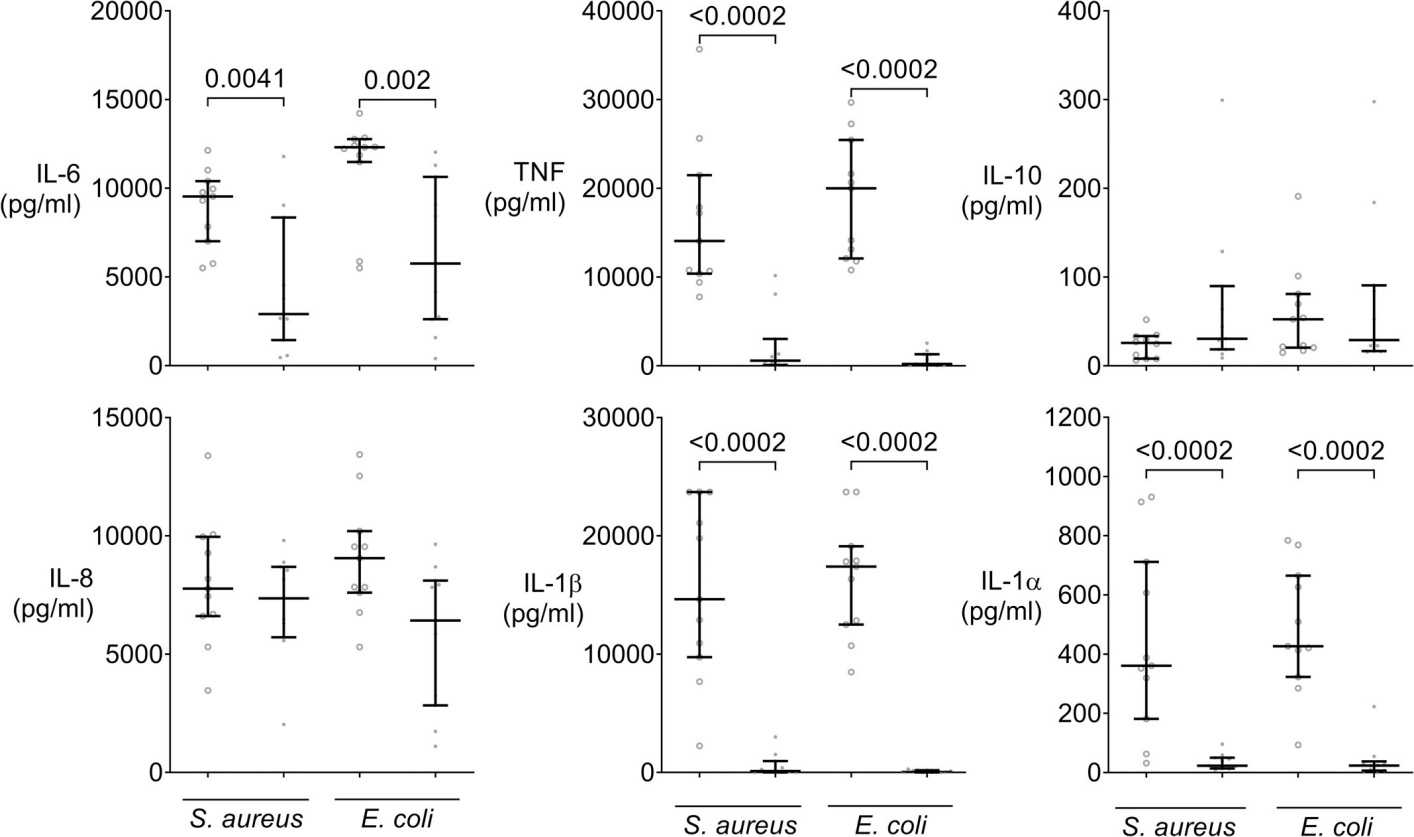
Depressed supernatant inflammatory cytokine concentrations in response to bacteria in early sepsis patients’ whole blood. Supernatant cytokine concentrations in whole blood assays of 11 sepsis patients (dots) sampled within 24 h of meeting Sepsis-3 criteria or matched 11 healthy volunteers (open circles) when incubated for 6 h either with heat inactivated *E*. *coli* or *S*. *aureus*. Dot plots with median and quartiles. Mann-Whitney tests with Holm-Bonferroni correction for multiple testing. Proinflammatory cytokine concentrations (IL-6, TNF, IL-1α, IL-1β) are markedly less in sepsis patients’ whole blood assays after exposure to bacteria, while there is no difference in anti-inflammatory IL-10 concentration.

Cytokine concentrations attained in response to *S*. *aureus* as well as to *E*. *coli* were markedly and significantly less in assays of sepsis patients than in those of healthy controls, as observed for IL-6 (median ratio: 3.3 & 2.1-fold), TNF (24 & 92-fold), IL1-α (15.8 & 18-fold), and IL-1β (132 & 527-fold), respectively, whereas no differences were seen for IL-10 and IL-8 (Fig 2). Taken together, inflammatory cytokine concentrations evoked in assays were depressed both in response to multiple TLR-ligands and inactivated bacteria in samples from sepsis patients when just meeting Sepsis-3 criteria.

### Depressed cytokine response to an *E*. *coli* LPS challenge associated to mortality

**Fig 3:**
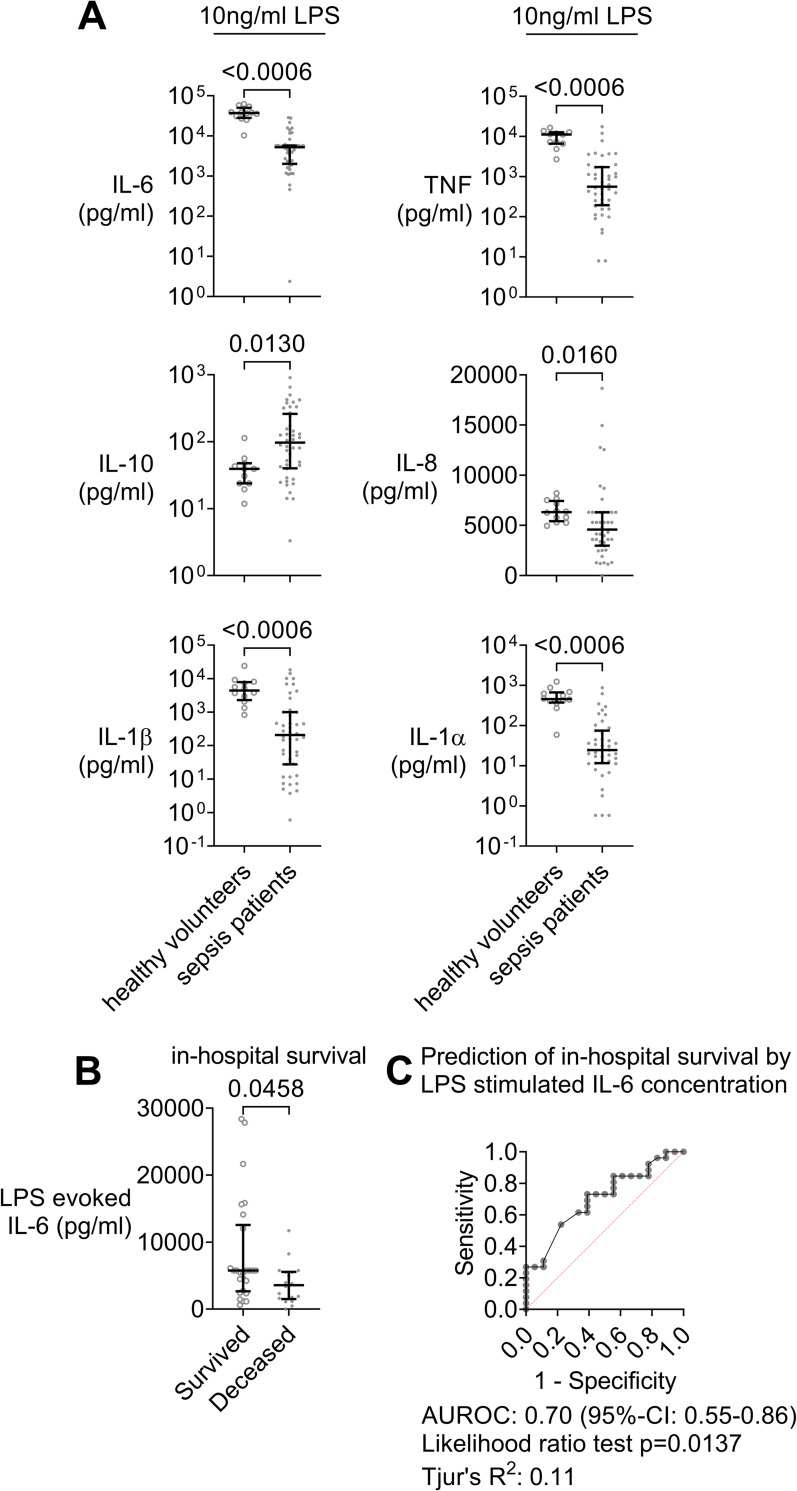
Diminished response to an *E*. *coli* LPS challenge of proinflammatory cytokine concentrations in whole blood assays of early sepsis patients and association of attained IL-6 concentrations with in-hospital survival. Whole blood from 44 sepsis patients (dots) sampled within 24 h of meeting Sepsis-3 criteria and 11 matched healthy volunteers (open circles) incubated for 6 h with 10 ng/ml LPS (*E*. *coli* O111:B4). **A** Comparison of supernatant cytokine concentrations. Dot plots with median and quartiles. Mann-Whitney tests with Holm-Bonferroni correction for multiple testing. **B** IL-6 supernatant concentrations in response to LPS of in-hospital survivors (n = 26) and non-survivors (deceased; n = 18). **C** Logistic regression of in-hospital survival on IL-6 supernatant concentration after 6 h of whole blood incubation with *E*. *coli* LPS as shown in a receiver-operator characteristic (ROC). An LPS challenge evoked much lesser supernatant proinflammatory cytokine concentrations in whole blood assays of sepsis patients than in controls and patients who did not survive showed a lesser response to the LPS challenge.

Supernatant inflammatory cytokine concentrations after 6 hours of whole blood incubation with *E*. *coli* LPS were markedly depressed in assays of septic patients compared to healthy controls when incubated for the same time (Fig 3A). This was seen for IL-6 (7.1-fold, p<0.0006), TNF (20-fold, p = 0.0006), IL-1α (18.6-fold), and IL-1β (21.5-fold, both p<0.0006). In contrast, anti-inflammatory IL-10 increased upon incubation when compared to samples from controls.

Stratifying the changes in IL-6 evoked by LPS in assays from sepsis patients there was a significant association of decreased IL-6 concentrations with in-hospital mortality (p = 0.046, Fig 3B). The receiver-operator-characteristic (ROC; Fig 3C) revealed an AUC of 0.7 for prediction of in-hospital mortality (95%-CI: 0.55–0.86; likelihood ratio test: p = 0.0137).

### Circulating P/DAMPs and inflammatory cytokine production in whole blood assays

**Fig 4:**
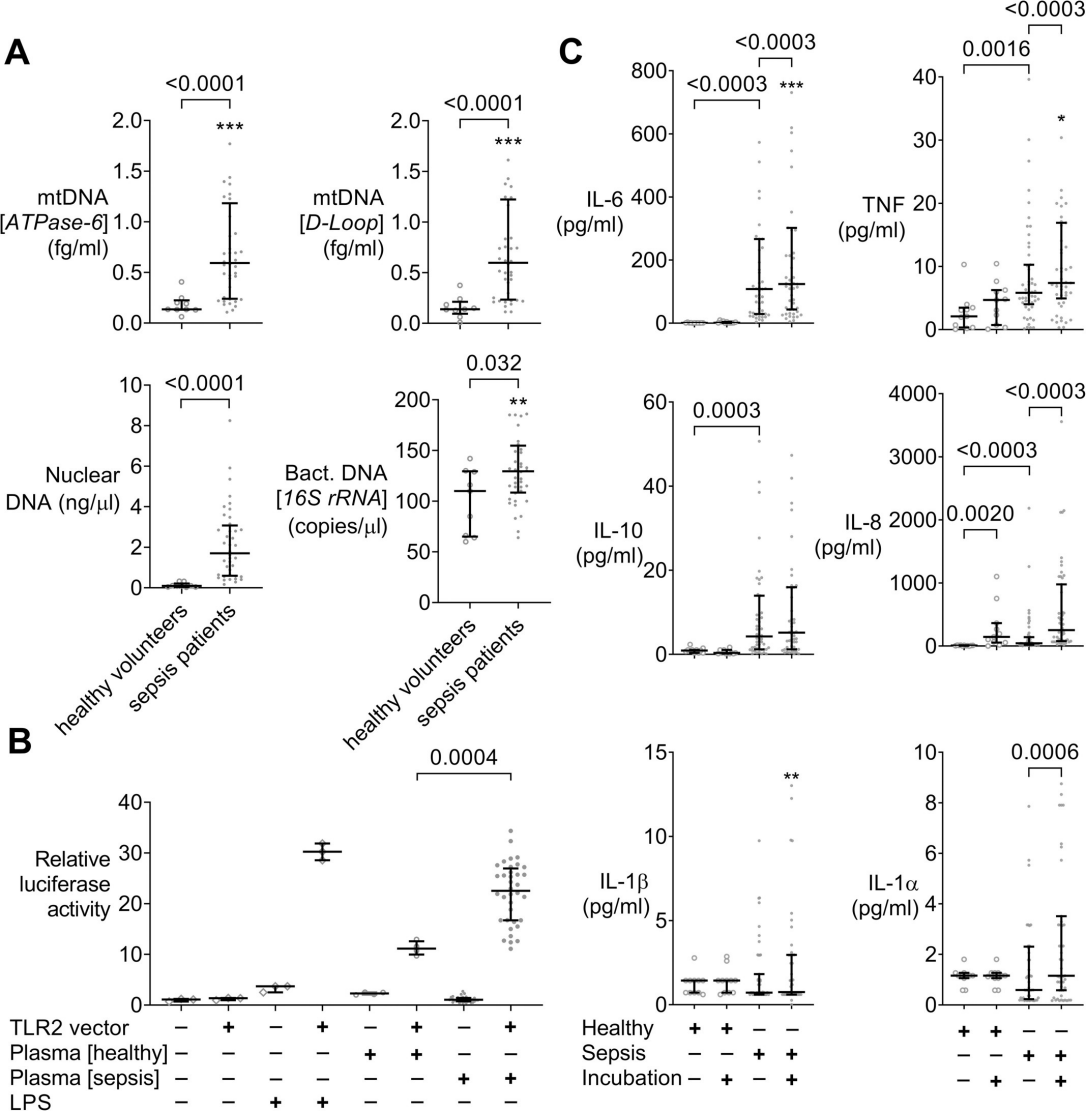
Circulating P/DAMPs, inflammatory activity, and *ex vivo* cytokine production of sepsis patients. **A** qPCR of plasma derived DNA preparations from 40 sepsis patients (dots) and 9 matched healthy volunteers (open circles) with primers towards host mitochondrial (mt), bacterial (bact.; r, ribosomal) coding sequences, and a human nuclear specific noncoding genomic DNA segment. Sepsis patients’ blood plasma contains greater concentrations of typical P/DAMPs. **B** TLR2 and NF-κB dependent luciferase reporter gene assay using transiently transfected HEK293 cells challenged for 16 h with plasma from 36 sepsis patients (dots) sampled within 24 h of meeting Sepsis-3 criteria or from 4 healthy volunteers (open circles). NF-κB dependent luciferase gene in all cells. Transfection with either TLR2 gene vector or empty vector as specificity control (TLR2 vector + / -). RPMI 1640 without or with autologous plasma with or without LPS (*E*. *coli* O111:B4) as positive and negative control (open rhombi). **C** Supernatant cytokine concentrations in assays of 51 patients with sepsis (dots) and 11 healthy controls (open circles) at baseline and following 6 h of incubation. As expected, inflammatory cytokine concentrations at baseline are greater in sepsis patients than controls. However, concentrations of IL-6 and TNF further increase upon 6 h of incubation in autologous plasma but not in controls. **A–C** *Data point out of axis limits. Dot plots with median and quartiles. Mann-Whitney or Wilcoxon matched pairs tests as appropriate with Holm-Bonferroni correction for multiple testing.

mtDNA concentrations (*ATPase-6* and D-loop) were markedly increased (p<0.0001 each) in samples from sepsis patients as were concentrations of nuclear (host genomic) DNA (p = 0.0001), and, to a lesser extent, bacterial DNA (p = 0.032, Fig 4A). As exemplified by a TLR-2 and NF-κB dependent luciferase assay (Fig 4B, p = 0.0004) and greater baseline cytokine concentrations (Table 1), inflammatory activity was increased in plain samples from sepsis patients compared to controls. Supernatant concentrations of the inflammatory cytokines IL-6 and TNF, even without additional stimuli, further increased over the 6 hour span of incubation in samples from septic patients but not in controls (Fig 4C), suggesting ongoing cytokine production *ex vivo*.

### Expression of cellular and soluble checkpoint molecules and the impact of checkpoint inhibition on cytokine concentrations

**Fig 5:**
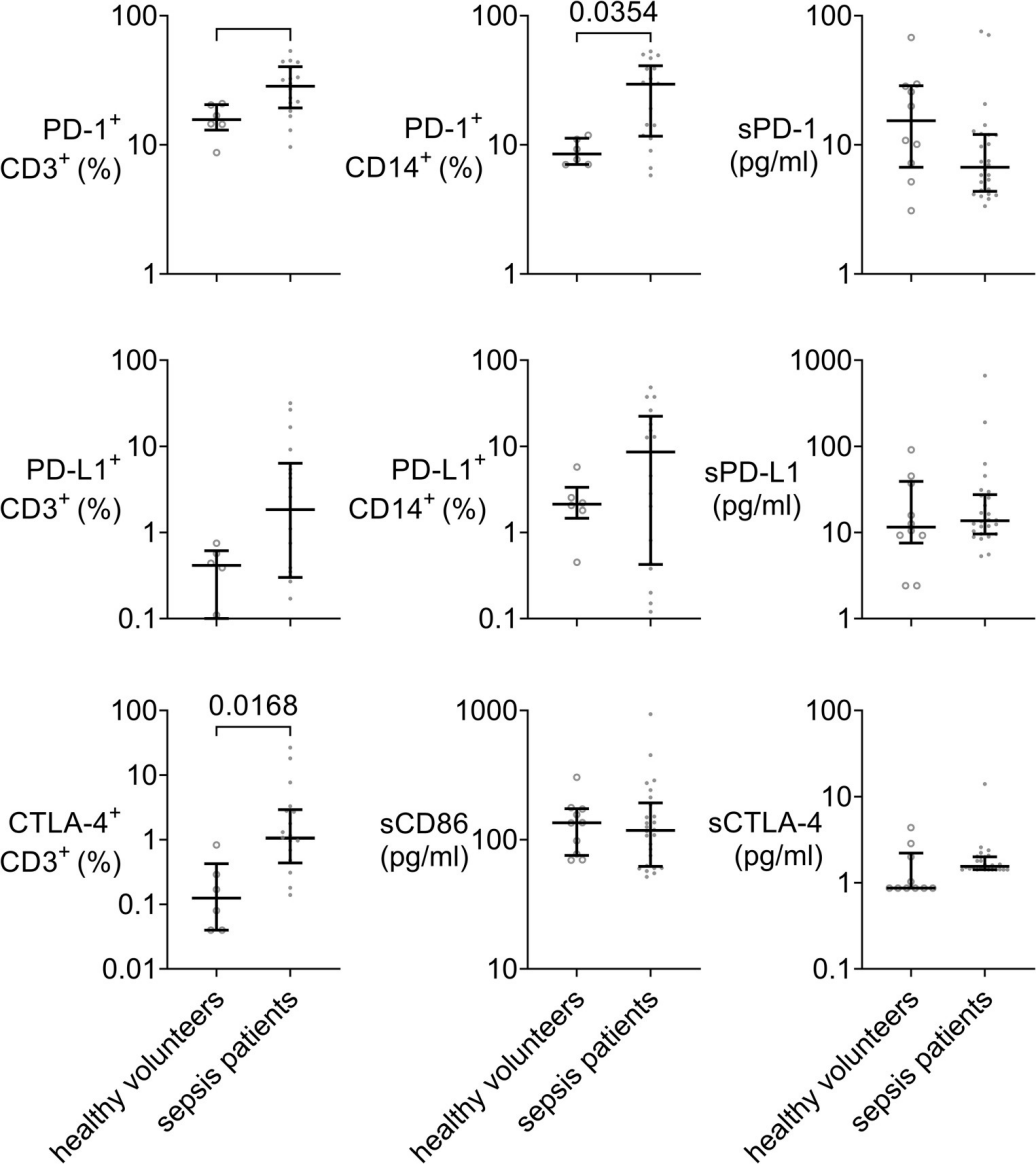
Greater checkpoint molecule expression in sepsis patients just meeting Sepsis-3 criteria. Percent of PD-1, PD-L1 or CTLA-4 positive cells of CD3 or CD14 positive cells (T-cells and monocytes, respectively) in the blood of 6 healthy volunteers (open circles) or 18 sepsis patients (dots) within 24 h of meeting Sepsis-3 criteria and concentrations of soluble PD-1, PD-L1, CTLA-4, and the CTLA-4 ligand CD86 in plasma. Dot plots with median and quartiles. Mann-Whitney tests with Holm-Bonferroni correction for multiple testing. Sepsis patients show significantly greater expression of cell-bound PD-1 and CTLA-4 immune checkpoint molecules than healthy controls, with similar soluble checkpoint molecule concentrations.

The percentage of T-cells and monocytes expressing PD-1 was significantly increased in samples from sepsis patients relative to healthy age-matched controls, whereas soluble PD-1 concentrations were not changed (Fig 5). CTLA-4 expression on monocytes was also increased (p = 0.017). Expressions of further cellular or soluble checkpoint molecules are provided in S2 Fig.

**Fig 6:**
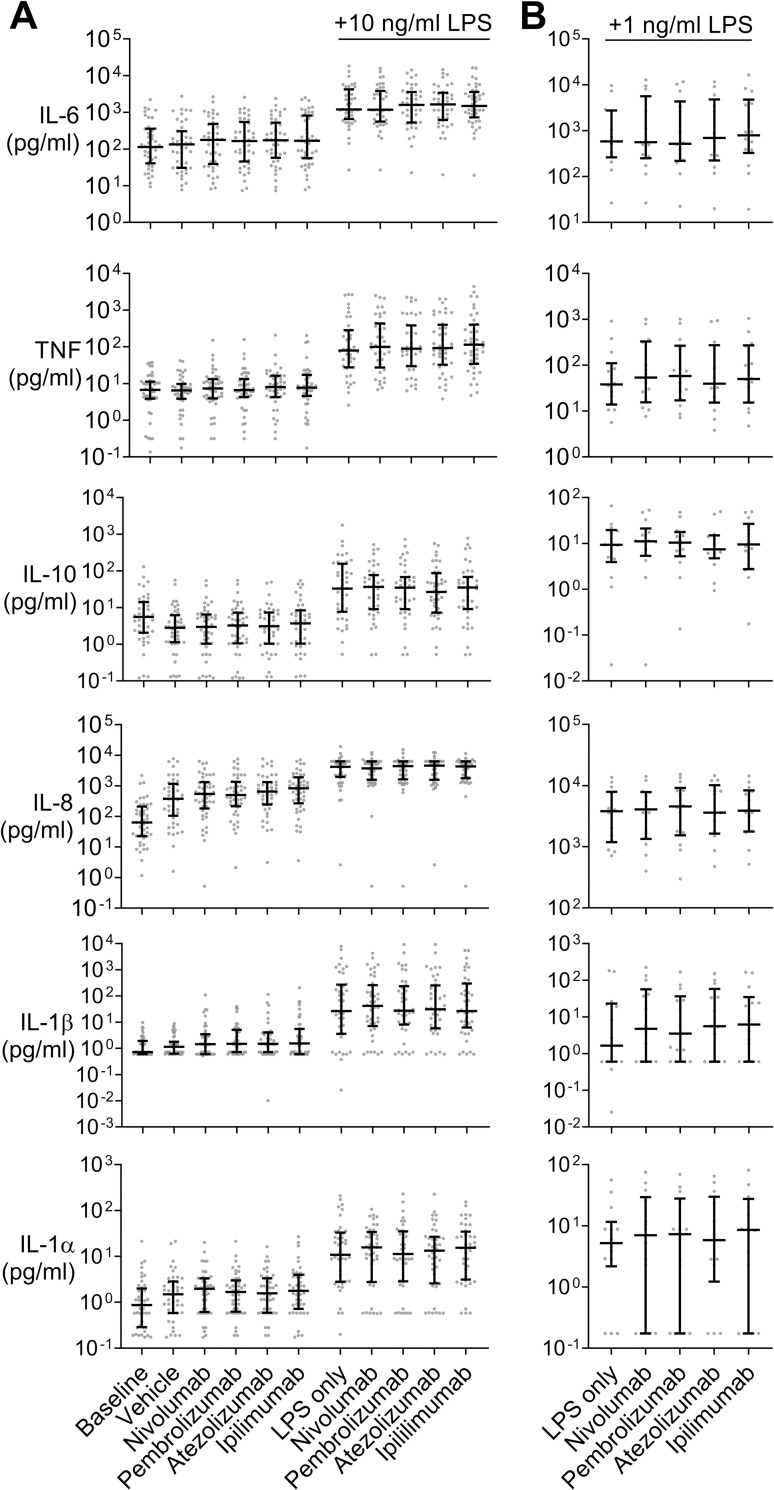
Immune checkpoint inhibition fails to increase cytokine concentrations in early sepsis patients’ whole blood assays. **A, B** Supernatant cytokine concentrations in whole blood assays of sepsis patients within 24 h of meeting Sepsis-3 criteria at baseline and when incubated with vehicle, LPS (*E*. *coli* O111:B4) only, the anti PD-1 mAb Nivolumab or Pembrolizumab, the anti PD-L1 mAb Atezolizumab, or the anti CTLA-4 mAb Ipilimumab with or without 10 (**A**; n = 44 patients) or 1 (**B**; n = 14 patients) ng/ml LPS. Addition of monoclonal antibodies at incubation start and of LPS 6 h thereafter. Overall incubation time was 22 h and supernatant cytokine concentrations were compared at the end of incubation. Friedman test with Dunn’s post-test for multiple comparison against the respective control (‘vehicle’ or ‘LPS only’, as appropriate). Neither blockade of PD-1, PD-L1, nor CTLA-4 increased the unstimulated or LPS stimulated cytokine production.

When samples were incubated with monoclonal antibody (mAB) checkpoint inhibitors directed at PD-1, PD-L1, and CTLA-4, respectively, there was no relevant or statistically significant increase in cytokine concentrations after 22 hours of incubation relative to controls, both in the absence and presence of 1 or 10 ng/ml LPS (Fig 6).

## Discussion

The present study, to our knowledge, is the first to provide a comprehensive view of blood-borne immunologic features of patients just meeting the hallmarks of sepsis, as defined by the Sepsis-3 criteria. While previous publications on septic immunosuppression mostly focused on late sepsis [22, 23] or amalgamated LPS-tolerance with broader septic immune tolerance [23], we observed a markedly mitigated cytokine production in response to multiple TLR-ligands (2, 4, and 7/8), to both Gram-positive *S*. *aureus* and Gram-negative *E*. *coli*, and to an *E*. *coli* LPS challenge within 24 hours of sepsis onset. At the same time, the hosts`blood revealed increased inflammatory cytokine concentrations further increasing on incubation and increased P/DAMP concentrations. Thus, functions of the innate immune system are depressed already very early in sepsis and this hyporeactivity occurs concurrently with inflammatory sepsis features, i.e., clinical symptoms and signs, and inflammatory cytokine production. With few exceptions [6], most previous work has described septic hyperinflammation and hypoinflammation as distinct and subsequent phases [1, 22]. Collecting data from different experimental setups, concomitant hyper- and hypoinflammation in sepsis has been discussed [13]. This study, however, demonstrates effects of both these elements of sepsis pathology at the same time and within the same model system. That this has a clinical impact is suggested by an increased mortality in patients with a lesser *in vitro* cytokine response to LPS. Despite such hyporeactivity and increased checkpoint molecule expression, however, immune checkpoint inhibition did not spark cytokine production *ex vivo* both in the absence or presence of LPS.

Although the understanding of sepsis mechanisms and supportive therapy have improved mortality, causal therapies appear not in sight [1, 24], possibly because translation from animal experiments is limited [8, 9, 25, 26] and it is difficult in practice to obtain and process samples in a timely fashion. While previous definitions of sepsis had been somewhat blurry the more recent Sepsis-3 definition has provided a common time anchor for the diagnosis of sepsis. Accordingly, we analyzed sepsis associated blood-borne immune responses with respect to a specific time, i.e., within 24 hours of first meeting Sepsis-3 criteria but not later. Rather than isolated cells we used the patients`whole blood that should incorporate all principle components of blood related inflammation, such as all leukocytes, causative pathogens, if still present, and the assortment of P/DAMPs and mediators prevailing in hosts which are known to stimulate TLRs [27, 28]. We chose this setup over cell isolation to model septic inflammation with as little manipulation as possible and including the complex interplay between all blood-borne cell types, their natural medium (plasma) and host plasma components [27]. While such systems might be preferable to cellular isolation when describing the net effect of complex systems or interventions, additional mechanistic insight might have been gained from repeating the experiments in more reduced model systems missing host plasma and other cell components, but this was beyond the scope of this study as sample volume available from critically ill patients limited the number of experiments we could perform per sample.

We found inflammatory supernatant cytokine concentrations of sepsis patients’ specimens to further increase *ex vivo*. This was likely mediated by increased concentrations of several P/DAMPs in the hosts`autologous plasma such as mitochondrial, genomic, and bacterial DNA, which were all markedly increased compared to controls, as exemplified by increased TLR2 agonistic activity. However, a maintained cellular program, previously activated *in vivo*, may have contributed. To our knowledge, such an ongoing *ex vivo* inflammation of whole blood has not been demonstrated before and might be an interesting model system for further studies on manipulating septic hyperinflammation. Furthermore, this set-up might in future studies allow a follow-up over the later course of sepsis.

At the time of this inflammatory response, however, inflammatory cytokine concentrations evoked in response to TLR2, TLR4, and TLR7/8 ligands were all markedly depressed compared to controls. This argues for a decrease in TLR receptor availability on cell surfaces, an inhibited TLR transduction process, and/or a decreased cytokine output due to other intracellular mechanisms, i.e., negative intracellular regulators [29], epigenetic regulation, or miRNAs. That cell function was not impaired by the assays themselves was confirmed by a spiking increase in cytokines after 6 hours of incubation when *E*. *coli* LPS was added to the assays. While we did not explore the molecular mechanisms contributing to this decreased reactivity to several TLR-ligands, depressed reactivity to ligands of multiple receptors of the TLR-family early in sepsis is interesting and may mechanistically relate to LPS tolerance [30–32].

Inflammatory cytokine responses to Gram-positive and Gram-negative bacteria were markedly depressed as well, possibly by the same mechanisms. This implies a broad inflammatory hyporesponsiveness reaching beyond individual signaling axes. Specifically, evoked supernatant concentrations of TNF, IL-6, IL-1α, IL-1β, and IL-8 were all substantially less in assays of sepsis patients’ samples, despite greater baseline concentrations and ongoing inflammatory activity when compared to controls. This was true both for *S*. *aureus* and *E*. *coli* which are common and prototypical strains related to sepsis [33]. Together, this indicates that at least some mechanisms in the defense against bacteria, i.e., cytokine secretion in response to bacterial encounters, are impaired very early in sepsis, i.e., already during the first day of sepsis.

The inflammatory cytokine response to an *E*. *coli* LPS challenge *ex vivo* was also much less in blood from sepsis patients than in control assays and this depressed LPS-evoked IL-6 production was associated with increased in-hospital mortality. A depressed cytokine response to LPS had previously been suggested as a potentially useful tool for monitoring in a report of 10 mixed surgical patients with sepsis of various duration [34].

Together, these data indicate an immune hyporeactivity present already within the first 24 hours of meeting Sepsis-3 criteria and occurring concurrently with inflammation. This strongly contradicts the canonical model of sepsis as an early hyperinflammation followed only later by hypoinflammation and immunosuppression, as proposed by the CARS concept [11]. A recent review updating present views of sepsis argued that a phase of “hyperinflammation” with organ dysfunction is followed later either by recovery, persistent inflammation, or immunosuppression and nosocomial infection, catabolism, and often death [22]. Our data indicate that at least some features of immunosuppression, notably the markedly decreased inflammatory cytokine secretion in response to multiple TLR-ligands and to bacteria occur already very early in sepsis (i.e., when just meeting Sepsis-3 criteria), concurrently with inflammation, and thus reveal a Janus-faced immune profile at this time. Such DAMP evoked reprogramming may also explain why patients with abacterial insults such as trauma, stroke, burns, or major surgery are prone to early infection [35–37].

Other reports have suggested early septic immunosuppression of blood cells [6] and splenocytes [5], although interpretation was hampered by concurrent corticoid treatment, use of isolated antigens, a different or wide-ranged timing of taking samples, and/or different sepsis inclusion criteria. Still others have cautioned that rather than designating such findings “immunosuppression”, or “immunoparalysis” one should more neutrally use the term “immune reprogramming” [13, 14] to account for potentially different mechanisms on the tissue level.

Regardless of terminology, sepsis patients are believed to enter an immune state fostering late infections [38–40]. Since one proposed mechanism contributing to septic “immunoparalysis” [4] is immune checkpoint molecule expression [41] this raised the question whether in sepsis immune checkpoint inhibition can unleash host defenses previously inhibited via immune checkpoint molecules [42], so as to increase immune activity and improve outcome. Cellular expression of CTLA-4, PD-1, or PD-L1 [43, 44] was increased in our patient cohort and we targeted these molecules to assess whether the cytokine response would increase. However, inflammatory cytokine concentrations did not change compared to controls with any of these checkpoint molecule antibodies, even when applied in concentrations similar to cancer therapy, and neither in the absence nor in the presence of different LPS doses as a second stimulus. Clinically, this finding is disappointing, in particular since we had found significant increases in concentrations of several checkpoint molecules in the septic patients`blood, as have others [45–49]. As checkpoint inhibition is considered a promising therapeutic modality in sepsis to counter immunosuppression and prevent nosocomial infections [50], this study adds relevant preclinical data regarding its potential effect. Whether these negative results with checkpoint inhibition correspond to a particular immune status, mirror unknown clinical features, or reflect immunosuppression mediated by yet untested checkpoint molecules remains unclear. Despite some *in vitro* and animal studies it remains unresolved whether ICI is effective in sepsis models or not [51–53]. Regarding their use in human sepsis, to our knowledge, no randomized controlled trial has been made beyond assessment of safety, tolerability, and pharmacokinetics [54, 55].

Since the early septic immune hyporesponsiveness appears to be unresponsive to PD1/L1 or CTLA4 inhibition, however, our findings question the potential use of checkpoint inhibitory treatments during early sepsis. Nevertheless, as the immune status may change during the further course of sepsis, timing or patient selection may be key and it remains to be assessed whether such approaches might be promising at later time points, only in specific patients, or under different circumstances. Furthermore, it is possible that the observed hyporeactivity might be alleviated by peptide receptor stimulation [56] rather than checkpoint blockade.

Sepsis is a heterogenous disease with diverse etiologies and host responses that in individuals may prevail to different degrees at different times. Accordingly, we strictly adhered to the recent Sepsis-3 criteria [10] and enrollment and taking samples within 24 hours. Nevertheless, despite using these criteria our data reveal some dispersion of clinical disease severity, P/DAMP concentrations, and checkpoint molecule expressions. Thus, while Sepsis-3 criteria are considered a step forward to unify recognition, definition of diagnosis, and epidemiology of sepsis, it appears less certain whether this definition helps to provide a common time anchor to fully understand prevailing mechanisms. Thus, assessing immune phenotypes prevailing in individual sepsis patients along their course may help to better understand these mechanisms and assays such as those used in this study may provide a toolbox to gain further insights.

### Limitations

Obviously, whole blood incubation *ex vivo*, while perhaps preferable to more reduced models, does not consider the interplay between blood and other body compartments or organs. Thus, our conclusions are limited to blood borne immunity. However, immunosuppression has been reported after a median of 8 days, albeit with a range of 1-195 days [5], in splenocytes of ICU patients dying from sepsis and multiple organ failure, suggesting that hyperinflammation and “immunosuppression” may or may not have a mutual time delay on the tissue level. While our clinical therapy followed established guidelines [57] and we did not apply high-dose corticoid therapy, we cannot rule out that other drugs may represent hidden confounders versus “natural” untreated sepsis which to study is not feasible ethically. Furthermore, as a tertiary referral center, we cannot exclude selection bias since our patients, although first meeting sepsis-3 criteria, represent a severely ill cohort and different results may have been observed with less severe sepsis.

## Conclusion

Within 24 hours of meeting sepsis-3 criteria, there is a markedly mitigated inflammatory cytokine production *ex vivo* in response to TLR2, 4, and 7/8 ligands as well as to bacteria prototypical for evoking sepsis such as Gram-positive *S*. *aureus* and Gram-negative *E*. *coli*. Thus, the immune system is depressed very early in sepsis and already at the same time as inflammatory key features of sepsis prevail. This Janus-faced profile contradicts the canonical late timing of immunosuppression and supports the presence of early immunosuppression already when patients are just meeting sepsis-3 criteria; yet the missing cytokine response to ICI despite increased expression of the corresponding checkpoint molecules appears to advice against immune checkpoint inhibition at this time point.

## Supporting information

S1 Table Microbiology of sepsis patients. (DOCX)

S1 File Raw data. (XLSX)

S2 File Flow cytometry details. (DOCX)

S1 Fig Concise summary of findings. (TIF)

S2 Fig Additional checkpoint molecule expressions in sepsis patients just meeting Sepsis-3 criteria. (DOCX)
