# Supplementary material for: Immune hyporeactivity to bacteria and multiple TLR-ligands, yet no response to checkpoint inhibition in patients just after meeting Sepsis-3 criteria
Source: PLoS One. 2022 Aug 18;17(8):e0273247. doi: 10.1371/journal.pone.0273247 (PMC9387870; doi:10.1371/journal.pone.0273247)
Supplement: S1 Table — (DOCX) [file pone.0273247.s001.docx]

**Table S1** Microbiology of sepsis patients

| **Suspected clinical focus of infection** | |
| --- | --- |
| Lung | 32 |
| Multiple foci / sources | 10 |
| Urogenital system | 6 |
| Other | 4 |
| Unknown at diagnosis | 7 |
| **Method of finding likely causative isolate^a^** | |
| Blood culture | 23 |
| Blood PCR (Septifast^TM^) | 10 |
| Culture of material from suspected focus | 5 |
| PCR of material from suspected focus | 3 |
| Urine antigen testing | 3 |
| No likely causative found | 24 |
| **Groups of likely causative pathogens** | |
| Gram-negative bacteria only | 13 |
| Gram-positive bacteria only | 7 |
| Mixed, Gram-positive and negative | 8 |
| Mixed, bacteria with other types of germs | 8 |

Microbiology results from 61 sepsis patients obtained at the time of

meeting sepsis-3 criteria. Data are number of patients.

^a^Some causatives were found by multiple methods.
