# Supplementary material for: Immune hyporeactivity to bacteria and multiple TLR-ligands, yet no response to checkpoint inhibition in patients just after meeting Sepsis-3 criteria
Source: PLoS One. 2022 Aug 18;17(8):e0273247. doi: 10.1371/journal.pone.0273247 (PMC9387870; doi:10.1371/journal.pone.0273247)
Supplement: S2 File — (DOCX) [file pone.0273247.s003.docx]

**S1 Supporting information Table 1.** Laser, filters, antibodies used for flow-cytometry.

| **Laser (Excitation)** | **Filter (Emission)** | **Channel Name** | **Fluorophor** | **Antibody** | **Cat. Nr. (Biolegend)** |
| --- | --- | --- | --- | --- | --- |
| 488 nm blue | 525/40 BP | FITC | AF488 | CD3 | 300320 |
| 561 nm yellow-green | 585/42 BP | PE | PE | CTLA4 (CD152) | 369604 |
|  | 610/20 BP | ECD | PE Dazzle 594 | PD1 (CD279) | 329940 |
|  | 780/60 BP | PC7 | PE Cy7 | CD14 | 301814 |
| 633 nm red | 660/20 BP | APC | APC | Tim-3 (CD366) | 345011 |
|  | 712/25 BP | APC-A700 | AF700 | CD19 | 302225 |
|  | 780/60 BP | APC-A750 | APC Fire750 | CD8 | 344746 |
| 405 nm violet | 450/45 BP | PB450 | BV421 | CD4 | 344632 |
|  | 525/40 BP | KO525 | BV510 | Zombie Aqua | 423101 |
|  | 610/20 BP | Violet610 | BV605 | PDL1 (CD274) | 329724 |

BP: Band pass.

**S1 Supporting information Table 2** Compensation matrix for flow cytometry

| **Autofl.** | **Channel** | **FITC [%]** | **PerCP [%]** | **APC [%]** | **APC-A700 [%]** | **APC-A750 [%]** | **PB450 [%]** | **KO525 [%]** | **V610 [%]** | **PE [%]** | **ECD [%]** | **PC7 [%]** |
| --- | --- | --- | --- | --- | --- | --- | --- | --- | --- | --- | --- | --- |
| 4,43 | FITC |  |  |  | 0,08 |  | 0,06 |  |  | 0,39 | 0,13 | 0,09 |
| 1,93 | PerCP | 1,8 |  | 2,7 | 4,37 | 0,07 |  |  | 6,11 | 13,62 | 33,61 | 0,96 |
| 0,45 | APC |  | 1,4 |  | 0,43 | 1,59 |  |  | 0,17 |  | 0,17 | 0,01 |
| 0,47 | APC-A700 |  | 18,7 | 38,17 |  | 0,94 |  |  | 0,09 |  | 0,11 | 0,04 |
| 0,33 | APC-A750 |  | 9,62 | 19,79 | 53,13 |  | 0,02 |  | 0,04 |  | 0,05 | 7,54 |
| 4,45 | PB450 |  |  |  | 0,01 |  |  |  | 2,78 |  |  |  |
| 0,00 | KO525 |  |  |  |  |  |  |  |  |  |  |  |
| 2,31 | Violet610 | 0,08 | 0,03 | 0,12 |  | 0,02 | 2,87 |  |  | 3,55 | 6,2 | 0,04 |
| 0,91 | PE |  |  | 0,02 | 0,02 |  |  |  | 15,04 |  | 18,44 | 0,96 |
| 1,52 | ECD |  |  | 0,17 | 0,17 | 0,05 | 0,03 |  | 42,09 | 57,59 |  | 0,62 |
| 0,50 | PC7 |  | 9,19 | 9,27 | 9,27 | 26,44 |  |  | 3,92 | 1,69 | 5,18 |  |

%; Percent signal compensation

**S1 Supporting information Fig 3**. **Gating strategy for flow cytometry.**





PBMCS of septic patients and healthy controls were thawn and subsequently stained. To exclude dead cells, PBMCs were stained with Zombie aqua, a DNA intercalating dye. „Zombie“– negative cells were forwardscatter-height vs. area (fsc-h/fsc-a) gated to exclude multiplets. We then chose a pan‑leukocyte gate in forwardscatter vs. sidewardscatter (ssc) instead of, e.g., a strict lymphocyte gate to also include granulated, i.e., activated, monocytes, or lymphocytes sometimes falling into the canonical granulocyte fsc/ssc area. To exclude other blood cells like remaining erythrocytes, we additionally performed pan-leukocyte staining with CD45 and analyzed positive cells for either expressing CD2 and CD3, CD19, or CD14, identifying these cells as T-cells, B-cells, or monocytes, respectively. Those were then analyzed for expression of PD-1, PD-L1, CTLA-4, Tim-3 (for T-cells), PD-1, PD-L1, Tim-3 (for monocytes), or PD-1, PD-L1 (for B-cells). Thresholds set using the respective antigen-negative populations in healthy volunteers’ samples (internal negative control) were used for healthy volunteers’ and sepsis patients’ samples to allow for direct comparison.
