# Supplementary figures and images for: Immune hyporeactivity to bacteria and multiple TLR-ligands, yet no response to checkpoint inhibition in patients just after meeting Sepsis-3 criteria
Source: PLoS One. 2022 Aug 18;17(8):e0273247. doi: 10.1371/journal.pone.0273247 (PMC9387870; doi:10.1371/journal.pone.0273247)

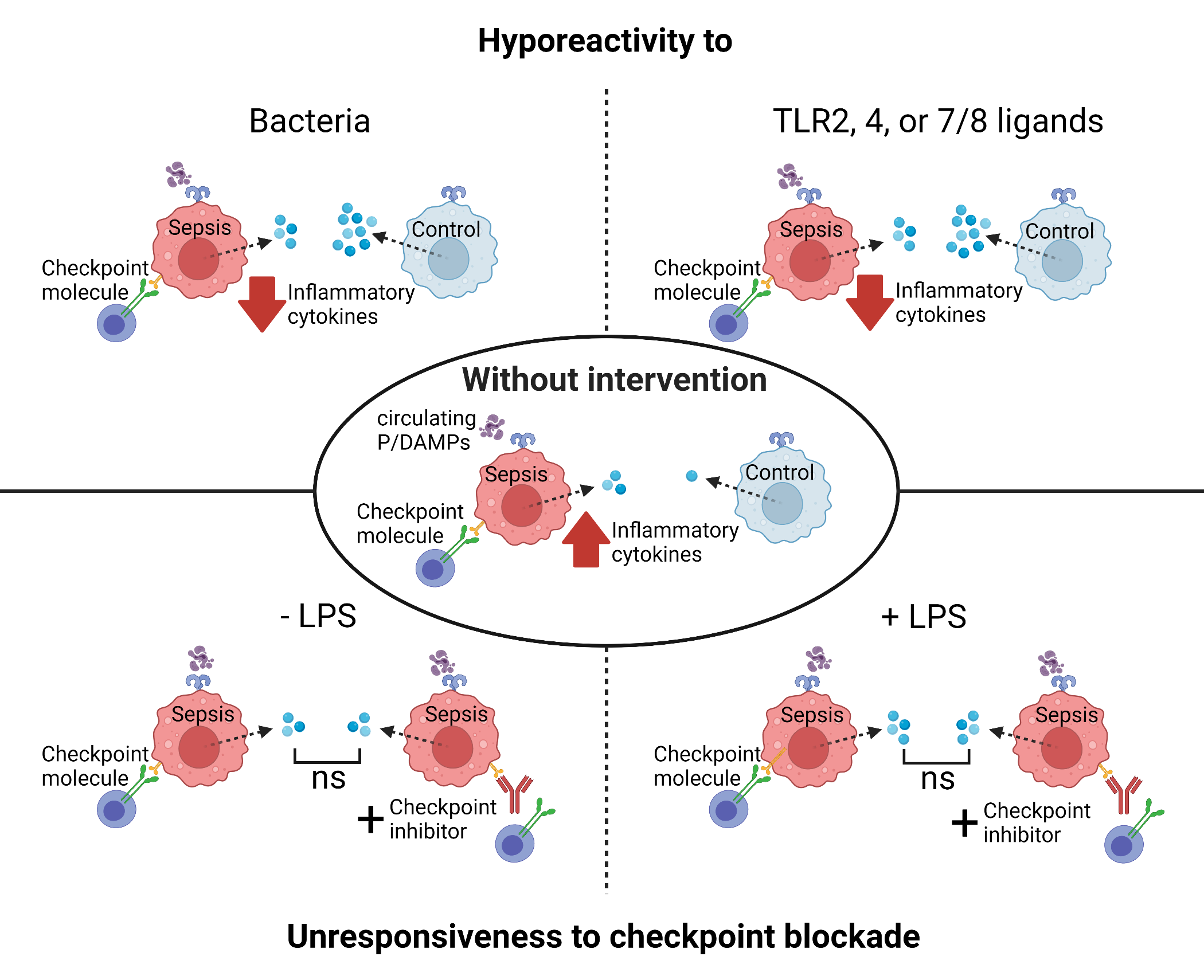

Supplement: S1 Fig — (TIF) [file pone.0273247.s004.tif]
