# Supplementary material for: Immune hyporeactivity to bacteria and multiple TLR-ligands, yet no response to checkpoint inhibition in patients just after meeting Sepsis-3 criteria
Source: PLoS One. 2022 Aug 18;17(8):e0273247. doi: 10.1371/journal.pone.0273247 (PMC9387870; doi:10.1371/journal.pone.0273247)
Supplement: S2 Fig — (DOCX) [file pone.0273247.s005.docx]

**S2 Fig. Additional checkpoint molecule expressions in sepsis patients just meeting Sepsis-3 criteria.**



**A** Tim-3 positive CD3 or CD14 positive cells (T-cells and monocytes, respectively), and PD-1 and PD-L1 positive CD19 positive cells (B-cells) in the blood of 6 healthy volunteers (open circles) or 18 sepsis patients (dots) within 24 h of meeting Sepsis-3 criteria.

**B** Concentrations of soluble CD25, CD137, Tim-3, Lag-3, and Galectin-9 in plasma of 26 sepsis patients and 10 healthy volunteers.

**A** – **B** Dot plots with median and quartiles. Mann-Whitney tests. * One data point out of axis limits.

Sepsis patients show significantly greater expression of some cell-bound and soluble immune checkpoint molecules.
